# “I Never Don’t Have Water Because I Collect Rainwater”: Domains of Water Insecurity and Their Sociocultural Correlates in an Indigenous Community of Northern Argentina

**DOI:** 10.1016/j.cdnut.2025.107519

**Published:** 2025-08-05

**Authors:** Sofia I Olmedo, Claudia R Valeggia, Cecilia Palavecino, Rafael Pérez-Escamilla

**Affiliations:** 1 Instituto de Investigaciones sobre Lenguaje, Sociedad y Territorio (INILSyT), Universidad Nacional de Formosa (UNAF), National Scientific and Technical Research Council (CONICET) Formosa, Buenos Aires, Argentina; 2 Department of Anthropology, Yale University, New Haven, CT, United States; 3 Member of Pilagá Community, La Bomba, Formosa, Argentina; 4 Department of Social and Behavioral Sciences, Yale School of Public Health, Yale University, New Haven, CT, United States

**Keywords:** water security, sociocultural correlates, Argentina, Indigenous people, HWISE

## Abstract

The lifestyles and worldviews of indigenous communities have long been deeply intertwined with natural resources, particularly water. These vital resources are now severely threatened by systemic social marginalization and the enduring impacts of colonization, further violating the human right to water access. Our primary objective was to assess the domains and correlates of water insecurity in a Pilagá community in Formosa, Argentina. This sequential exploratory mixed-methods cross-sectional study, conducted in 2023, involved data collection from Pilagá households representing 59 family clusters, covering all family units in the community. We used a prevalidated Household Water Insecurity Experience survey. Qualitative data were gathered through semistructured interviews and participant observation. The average age of participants was 36.8 ± 12.7 y, with most being women, who primarily handled the task of fetching water. Water insecurity was prevalent, affecting 62% of households, most of which depended on well pumps. The most serious concern associated with water was the lack of long-term stability. Through an ecologic model, we identified multiple interrelated contextual variables, revealing that shifts in one area (geographic, capitalistic market, water policies, and infrastructure policies) had ripple effects across others. Key correlates included water sources, cultural perceptions of water, resource distribution, and social dynamics around water. The Pilagá community confronts pervasive water insecurity within a challenging and evolving socioecologic landscape.

## Introduction

Water security is a fundamental human right, as affirmed by the United Nations General Assembly in 2010, which has explicitly acknowledged that access to clean drinking water and sanitation is essential for the realization of all human rights [1]. Today, this fundamental right remains an outstanding social debt with vulnerable populations. Latin America is currently facing a crisis in water supply and sanitation services, with two thirds of the population lacking access to services that meet international standards [2]. In Argentina, 7 million people lack access to safe drinking water and nearly 20 million do not have access to proper sanitation services [3]. This crisis is especially severe in rural areas, small towns, and urban slums, where people face persistent barriers to having access to safe water and sanitation [2].

Although water security is recognized as a human right, many Latin-American countries still lack robust policies for ensuring access to safe drinking water. For instance, Argentina has yet to implement a comprehensive water law. Although Argentina’s National Directorate of Drinking Water and Sanitation provides strategic guidance and funding through the Federal Water and Sanitation Plan, provincial institutions have the responsibility of water policy implementation at the local level [3,4]. Our study was conducted in the northern province of Formosa, one of the most neglected regions of Argentina, consistently ranking highest (together with the province of Chaco) in unfavorable social and public health indicators such as maternal and infant mortality, poverty, and limited access to essential services [5]. In Formosa, water management is led by the Water Coordinating Unit and the Provincial Service of Drinking Water and Sanitation [6]. These entities are responsible for ensuring water access both in the provincial capital—through piped water networks—and in rural and peri-urban areas, primarily through tanker truck distribution [6]. The coordination between the national and provincial levels occurs through project planning, financing mechanisms, and technical support, whereby the Federal Plan aligns with local needs and capacities. This multilevel governance model seeks to ensure that national investment priorities effectively address provincial disparities in water and sanitation access, particularly in underserved regions such as the interior of Formosa [3].

**FIGURE 1:**
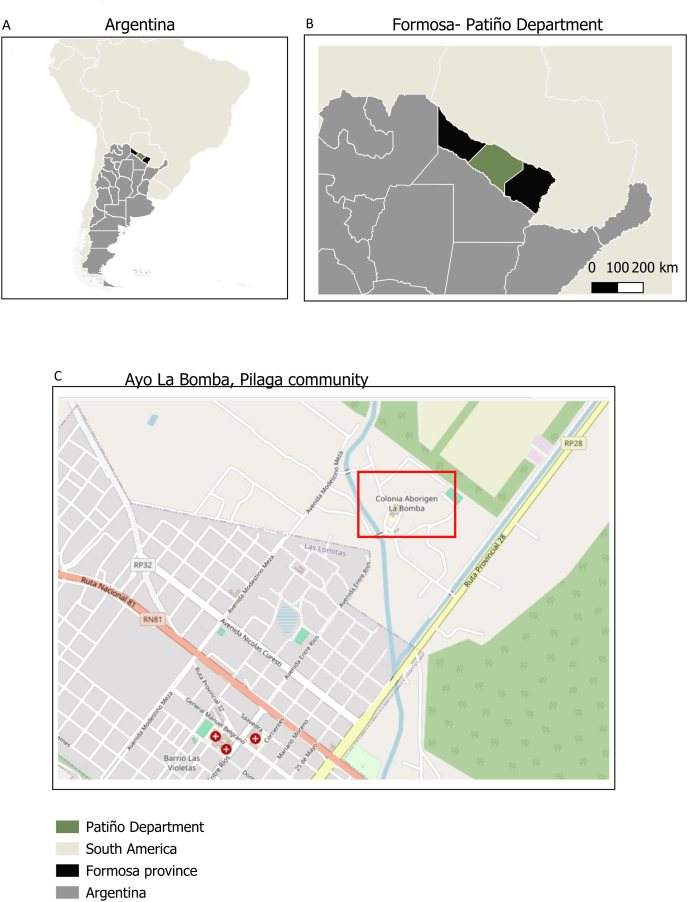
Study area. Location of the Ayo La Bomba, Las Lomitas, Formosa, and Argentina: (A) country and state and (B) Ayo La Bomba territory. Country and state basemaps source: IGN (National Geographic Institute) www.ign.gob.ar/NuestrasActividades/InformacionGeoespacial/CapasSIG (25) available under open license. Ayo La Bomba territory basemap source: U.S. Geological Survey. (n.d.). USGS EROS Archive - Aerial Photography - Aerial Photo Single Frames. U.S. Department of the Interior. Retrieved July 30, 2025, from: https://www.usgs.gov/centers/eros/science/usgs-eros-archive-aerial-photography-aerial-photo-single-frames?qt-science_center_objects=0#qt-science_center_objects (QGIS, version 3.34.13).

Our study focuses on a Pilagá community in Formosa. The Pilagá originally inhabited the Pilcomayo River area before it became the border between Paraguay and Argentina. Historically, they were seminomadic hunter-gatherers, but today they reside in 23 settlements across the Patiño and Bermejo Departments of Formosa. The study focused on communities situated along Route 28 (Figure 1). Their water supply is directly influenced by the Pilcomayo River and Bañado La Estrella. From the 1920s to the 1990s, repeated obstructions in the river channel along the Argentina-Paraguay border caused Bañado La Estrella to expand significantly [[7], [8], [9]]. Since the 1990s, the construction and improvement of Provincial Route 28 have triggered a cascade of socioenvironmental challenges, including flooding, water scarcity, and pollution [10,11]. Alongside the route, a water channel was built on its western side (upstream) to connect the wetlands with Las Lomitas, despite the region’s unfavorable southern slope [8]. As foreseen by residents and experts, neither the diversion channel nor the bridge met the government's expectations. The channel quickly collapsed, becoming obstructed with sediment, and thousands of hectares upstream were flooded [9]. The flooding caused extensive economic losses for both the Pilagá and the other populations, including damage to cultivated fields, livestock, and forest resources [8,11]. In the Pilcomayo, pollution, recurrent mass fish die-offs, and sudden overflows have become closely linked to water insecurity [8,12].

Stable access to water is vital to sustaining the core values and traditions within indigenous communities [13], for this reason, evaluating the lived experience of accessing and utilizing safe water is a complex process that requires an integrated perspective, taking the broader context into account.

### Water security and indigenous populations

Household water insecurity is defined as the inability to access and utilize sufficient, reliable, and safe water for well-being and a healthy life. This insecurity can stem from issues related to excessive, insufficient, or poor-quality water [14,15]. Household water insecurity occurs when any domain of water security (availability, accessibility, use, and stability) is compromised. Stability (or reliability) includes availability, access, and use [14,16]. Water insecurity can arise not only from the lack of availability of the resource but also from poor management, insufficient funding, or inadequate infrastructure, highlighting the multifaceted nature of the condition [13,17]. The absence of piped water in homes does not necessarily indicate a lack of water security, but it does increase vulnerability to it. Likewise, infrastructure for piped water per se does not guarantee water security. Indigenous peoples, who are often constrained by socioeconomic hardships, face significant challenges in affording water or making the necessary investments to secure reliable water services [18]. In addition, they are disproportionately affected by climate change and dispossession of land [19], and the enduring impacts of colonial exclusion [17] due to their deep reliance on natural resources in their territories.

Water shortages can arise from a complex interplay of factors, including climatologic causes such as prolonged droughts, hydrologic issues such as excessive groundwater extraction, and sociopolitical forces such as water mismanagement and price inflation that restrict access for vulnerable populations [20]. In addition to scarcity, numerous factors can render water unsafe or entirely inaccessible. Natural contaminants such as pathogens, arsenic, fluoride, and salt intrusion pose serious health risks. Meanwhile, human-made pollutants further degrade water quality, including perfluoroalkyl and polyfluoroalkyl substances, fertilizers, and microplastics [20]. Infrastructure itself can be a source of contamination, with lead leaching from aging pipes and pharmaceutical and personal care residues seeping into water supplies [20,21]. These persistent pollutants pose serious risks to human health, particularly for vulnerable groups such as indigenous communities, and also degrade ecosystems, further intensifying the water crisis.

In contrast, water availability refers to the physical presence of water and its distribution, whereas access concerns the ability to obtain and control it as a commodity. Usage encompasses water safety practices, needs, and associated costs [22]. Researchers emphasize the importance of understanding the lived experience of water insecurity alongside availability and access [23]. The lifestyles and worldviews of the *pueblos originarios*—the term used in Argentina for indigenous peoples—are deeply interwoven with natural resources, especially water [24]. The dispossession of land and water not only disrupts indigenous relationships and responsibilities such as conflicts arising from restricted access to natural water sources due to privatized land but also devastates these communities’ capacity to attain “living well” (*buen vivir*). For these communities, living well is fundamentally tied to access to clean water, which is vital for sustaining both their ecosystem physical life and cultural identity [19]. Indigenous peoples view water not as a mere resource to be exploited or managed in isolation but as an integral part of an interconnected web that encompasses all natural resources and living beings. Their water management practices are grounded in a holistic vision of the land, centered on deep respect and stewardship of rivers, springs, lakes, and wetlands [9].

This study seeks to assess the domains of water insecurity and its correlates in a Pilagá community in Formosa, Argentina. In our study, it is the historical and socioenvironmental context of the Pilagá community, recognizing that for Indigenous Peoples, water is not merely a resource but holds deep cultural, spiritual, and life-sustaining significance [13].

## Methods

### Study population and design

This cross-sectional sequential exploratory study, reported according to the Mixed Methods Appraisal Tool, was conducted from January to July 2023 in a Pilagá community in Formosa, Argentina. This design enabled data triangulation and a deeper exploration of the correlates of water insecurity than a single method would allow, offering a more comprehensive understanding of its social and material contexts than quantitative data alone. The qualitative methods deepened our understanding of the quantitative findings by revealing how individuals experience water insecurity and how they perceive issues related to water access and usage in their daily lives.

We focused on La Bomba, a peri-urban Pilagá community located 300 km west of Formosa’s capital, which comprises 113 family units. The study included 59 family clusters, reflecting the community’s strong kinship networks, where multiple families share land while maintaining separate households. A nonprobabilistic snowball sampling method was used to recruit heads of households and community leaders, leveraging social networks to capture community beliefs and perspectives. We used this well-understood sampling method to enhance credibility by drawing on relationships among key actors [25].

### Data collection methods

Data collection was conducted by SO, with CP, and a bilingual Pilagá community member, who assisted with recording and interpreting, particularly for the ethnographic component. Each of the 59 participating family clusters was visited at least 3 times. During these visits, the validated Spanish version of the Household Water Insecurity Experiences (HWISE) scale was administered to the head of household, who was typically the main caregiver and water decision-maker. In addition, we asked each participant about household water sources and their preferences for drinking water.

The HWISE Scale consists of 12 simply phrased questions that take ∼3–5 min to administer. Although this instrument has not yet been used in this population, it has demonstrated good internal consistency and cross-cultural applicability in diverse low-resource settings [16]. Briefly described, the scale measures multiple domains of water insecurity, including whether there is enough water for all household needs (*Adequacy*), the consistency and predictability of water availability (*Reliability*), the ease with which water can be obtained (*Accessibility*), and the quality and potential health risks associated with the water available (*Safety*) [16].

Quantitative data collection preceded the qualitative phase of the study. After completing the HWISE survey, a subsample of participants was selected to participate in semistructured interviews conducted by the first author with those participants who expressed interest in further elaborating on the topic. Each interview lasted between 45 and 60 min and followed an interview guide focused on drinking water sources, preferences, and strategies for obtaining water during periods of scarcity. The guide was developed by the first (nutritionist) and second authors (human biologist) and pretested by the third author (Pilaga community health agent). After each day of fieldwork, the interviews were transcribed and analyzed to determine when information saturation had been reached.

In parallel, ethnographic observation was carried out with 59 participant households. The first author systematically visited each family cluster to document water-related practices including infrastructure, water facilities, sources of supplies (e.g., natural sources, small shops, stored water, and trunk tank) and the geographic distribution of households. These observations were recorded as detailed field notes and complemented the data obtained through interviews and the HWISE scale.

### Quantitative data analysis

The HWISE scale scores were calculated by summing the responses across 12 items, with values ranging from 0 to 36—higher scores indicating greater water insecurity 4 response categories were used: "never" (0 times) was scored as 0; "rarely" (1–2 times) as 1; "sometimes" (3–10 times) as 2; and "often" or "always" (>10 times) as 3. We selected cut-points in water insecurity scores to establish 4 ordinal categories: no-to-marginal (0–2), low (3–11), moderate (12–23), and high (24–36) water insecurity [26]. In the original validation, these categories were monotonically associated with increasing odds of reporting water dissatisfaction and helped to differentiate the breadth of water insecurity across populations with heterogeneous water insecurity experiences and frequencies. As recommended in the User’s Manual, households were not assigned a score if participants responded with “I don’t know” or “not applicable” to any item [16]. Following these guidelines, households with a score of 12 or higher were classified as water insecure [16]. Cronbach’s alpha for the 12-item scale was 0.81, indicating good reliability in this study setting. Additionally, we documented the proportion of participants’ households accessing each self-reported water source and the participants’ preferred drinking water sources.

### Qualitative data analysis

**FIGURE 2:**
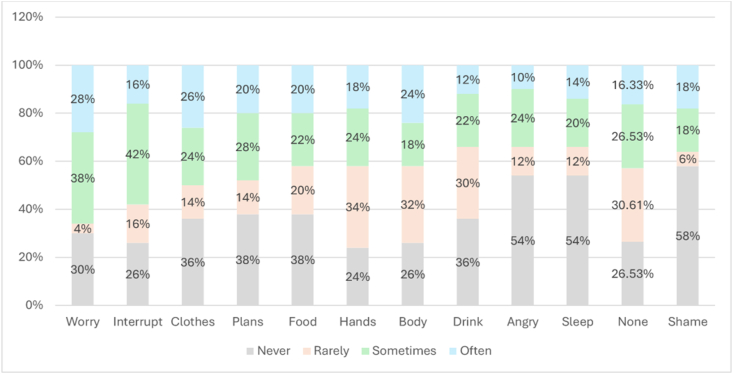
Frequency of 12 HWISE items reported by the Pilagá people in Formosa, Argentina. HWISE, Household Water Insecurity Experiences.

Qualitative data analysis was informed by transcribed field notes and interviews, which were analyzed using word processing software. Data collection via interviews stopped after reaching saturation at *n =* 10 interviews [26]. Content analysis was then conducted to identify emerging thematic categories related to cultural patterns, that is, cultural practices, beliefs, and daily routine that correlate with how participants perceive, access, and manage water. The first author initially analyzed the transcripts using a word processing program to organize and code the data, generating preliminary codes and thematic categories. A predetermined codebook was collaboratively developed by the authors, based on a combination of deductive codes derived from the HWISE framework (e.g., availability, access, reliability, stability, use, and water preferences) and inductive codes that emerged from the participants’ narratives. Subsequently, we utilized IRaMuTeQ software to produce word cloud images that visually represented a similarity analysis, allowing us to observe relationships among highlighted words, their positioning within the diagram, and the connections between them in a tree structure (Figure 2). This analysis contributed to identifying salient themes in participants’ narratives supporting our objective of understanding the correlates of water insecurity.

*Responsible conduct of research*: the study, which was approved by the Yale Institutional Review Board (Protocol #14456), involved initial visits to explain objectives, distribute informational sheets, and invite participation. Acknowledging the Indigenous cultural practice of prioritizing verbal agreements, consent was documented as a voice recording. Interviews were conducted during follow-up visits with consented participants.

## Results

### Population characteristics

The participants had a mean age of 36.8 ± 12.7 y, with ages ranging from 17 to 70 (Table 1). Most participants were women, who bore the primary responsibility for fetching water due to the community's lack of access to a public drinking water service. Ethnographic field notes revealed that families depended on monthly cash transfers from the government's social support system as their primary means of subsistence. Additionally, men supplemented household income through informal work such as bricklaying and gardening, whereas women contributed by producing and selling handicrafts and by engaging in domestic work.

**TABLE 1:** Socioeconomic and demographic characteristics of participants from a Pilagá community in Formosa, Argentina (*N =* 59).

Variable	Category	Percentages
Gender of participants	Female	85
	Male	15
Age	17–25 y old	22
	26–35 y old	34
	36–50 y old	25
	>50 y old	19
Education level	Finished some or all elementary school	42
	Finished some high school	29
	Completed high school	17
	Attended postsecondary school	12

The houses in the community were built from bricks, either provided by the government or self-constructed, contrasting with the traditional mud and palm huts of the more rural Pilagá villages. The construction bricks were either provided by the government or built by the residents themselves, standing in stark contrast to the traditional mud and palm huts in the more remote Pilagá villages. The houses in our study’s lacked potable water facilities and relied on basic latrines.

### Experiences of water insecurity within the community

As assessed by the HWISE scale, 62% of households reported experiencing water insecurity, which was classified at the following level of water insecurity: *1*) 38% as low water insecurity; *2*) 48% as moderate water insecurity, and *3*) 14% as high water insecurity. The overall mean HWISE score was 15.0 (95% CI: 12.8, 17.2), out of 36 possible points, which corresponds to moderate water insecurity.

The HWISE scale assessment of household water insecurity highlighted the emotional and behavioral impact of water insecurity. Approximately 42% of respondents indicated that their water security—and, by extension, their everyday routines—were “sometimes” disrupted because of water-related challenges. Although between 24% and 38% of participants reported that specific domestic activities, including laundry, household planning, cooking, handwashing, and bathing, were “never” affected, the overall findings suggest that water insecurity prompted notable adjustments in daily routine, such as cooking, washing, and cleaning, among a substantial proportion of the population. In terms of negative emotions, only one-third of households reported never feeling worried about water availability, whereas about half said they never experienced anger or embarrassment due to water scarcity. Although more than half of participants reported never going to bed thirsty, only one-third indicated that their household rarely or sometimes had no usable or drinkable water available at all (Figure 2).

### Water sources and accessibility

**FIGURE 3:**
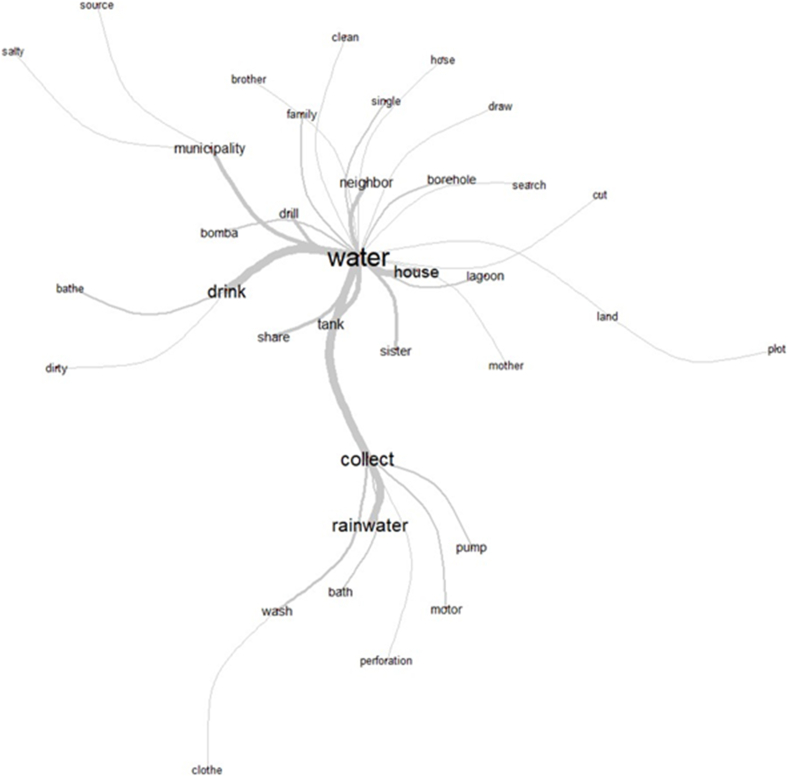
Words related to water security from the perspective of the Pilagá community in Formosa, Argentina (IRaMuTeQ, version 0.7 alpha 2).

In the absence of public drinking water services, households relied on 2 primary water sources: 79% accessed water through a well drilled on their property, requiring the use of a pump, whereas the remaining households obtained water from a municipal tanker. Participant observations and interviews revealed that the most frequent word associations were “water-house-drink” and “water-house-motor” (Figure 3), as nearly all family units owned ≥1 water pump. Although, due to the high cost of drilling, not all families could afford to secure a safe water source in their homes, some families collectively financed the drilling of shared wells on communal land to ensure access to drinking water.

Despite investments in infrastructure, both physical and economic barriers continued to restrict access to water. Although rainwater collection was a common practice, occasionally by choice, it was more often a response to need. The relationships “water-municipality” and “water-catchment-rainwater” emerged as secondary water sources. Participants reported storing water in tanks and contacting the municipality when their supply ran low. Many also collected rainwater even if they had access to water from a pump or municipal supply.

### Perception of water quality

Water quality was a concern both inside and outside the community. In a nearby village, 5 km from La Bomba, bottled water was sold in all local shops because the main’s drinking water was considered unreliable. Within La Bomba, a small shop sells bottled water and other beverages, primarily sodas. However, among all participants (*n =* 59), only 1 person mentioned purchasing bottled water from the village when they could afford it. The rest reported alternative ways of obtaining water, such as fetching water from the lagoon, collecting rainwater, or relying on neighbors or relatives.

Participants described the water as having an unpleasant taste and appearance, and reported instances of contamination, including bleach-like odors and visibly dirty or discolored water. Notably, during periods of scarcity, concerns about water quality often became secondary. When physical access to water was limited, the priority shifted to obtaining any water, regardless of whether it was safe for drinking or not. Regarding drinking water preferences, 45% of participants favored water from pumps, whereas 38% preferred rainwater. Additionally, 9.5% expressed a preference for both pump water and rainwater, whereas only 4.8% favored water from municipal tanker tanks, which was frequently described as having a bleach-like taste. A small number of participants preferred bottled water (2.7%), though it was often difficult to obtain due to physical and socioeconomic constraints.

### Infrastructure, maintenance challenges, and policy

As previously mentioned, the Pilagá community lacked access to the public drinking water network that supplies the nearby town of Las Lomitas. The neighborhood was named “La Bomba” after the water pump that provides water to local families. In the past, before wells were drilled, the community relied on communal pumps, where women fetched water for their households. Today, in addition to water from boreholes and municipal sources, participants also reported utilizing other water sources, including rainwater, a nearby water mill, and surface water from the lagoon.

*“If I don't have clean water to cook or drink, I use rainwater from the storage tank” (woman, 24 y old)*

*“I never don’t have water because I collect rainwater” (woman, 42 y)*

*“Their primary water source is from the municipality but when they didn't have water they got it from the mill” (fieldnotes)*

*“When I didn't have drinking water, I looked for the lagoon” (woman, 28 y old)*

In terms of water policy, the secondary source of water was the weekly supply provided by the municipality. Nonetheless, participants reported several issues, including a strong bleach taste in the water, insufficient cleaning of storage tanks, and challenges in maintaining water quality due to limited availability. In both municipal water storage and rainwater collection systems, a notable lack of tank cleanliness has been observed. Some participants reported cleaning their tanks infrequently, citing the persistent scarcity of water as a primary barrier to regular maintenance.

Although a windmill-powered pump was available as an extra water source in the community, it was used by only a few participants due to its reliance on wind conditions and long distance from their households.

### Water resource allocation and community tensions

In response to water scarcity, a third of households with access to water from drilled wells shared their supply with 1 to 5 other households. This sharing was made possible by the proximity of households, many of which were part of the same family and shared land. When participants had cash from temporary work or social protection subsidies, they typically did not purchase water. Instead, they prioritized spending on food and relied on alternative water sources, such as asking neighbors or collecting water from the lagoon.

The water-sharing network underscored the social and political dynamics within family structures, ensuring that essential resources such as water were distributed through long-standing communal sharing practices. However, tensions have emerged between the moral responsibility to share and the necessity of conserving resources for one’s own household. This conflict, driven by water scarcity, has led to disputes among family members.

*Her water source is from the municipality that comes once a week. She fights with her sister for water when there is no water (fieldnotes).*

Socioeconomic factors heavily influenced these outcomes, particularly the high costs associated with installing wells equipped with water pumps. The expense for such installations equals or even exceeds the minimum required to sustain a typical family of 4, prompting multiple families to pool resources for shared drilling. Additionally, bottled water is prohibitively expensive, with a 20-L drum costing 1% of the basic basket and lasting only a week for a typical family. This results in an expenditure of 4% of the basic basket solely for drinking water each month. Given that social policy protections provided families with an income amounting to just 15% of the basic basket, they were forced to prioritize purchasing food.

### Gender dynamics and water

In our study, we observed that the responsibility for ensuring water availability in the home falls on the women of the Pilagá community. They were tasked with bringing water from drilled wells or fetching it from the lagoon and other water sources. These women were deeply concerned about water insecurity and continuously strategized on how to secure this vital resource.

The role of women in water management was just one aspect of their broader responsibilities within the household, which also include food procurement and preparation. In this community, the link between food insecurity and water insecurity was significant, with 44% of households reported that they “sometimes” or “often” adjusted their diets due to water scarcity. Women were the primary decision-makers when it came to determining what to eat, who will eat, and how to obtain food. Water shortages, particularly for drinking and cooking, became more severe during the summer months, worsened by droughts and frequent power outages. Although the entire region faced water shortages in the summer, the situation in the Pilagá community was especially dire. Despite this, some women asserted that they “have water,” as they relied on the lagoon as an alternative source.

### HWISE domains in Pilaga community

**FIGURE 4:**
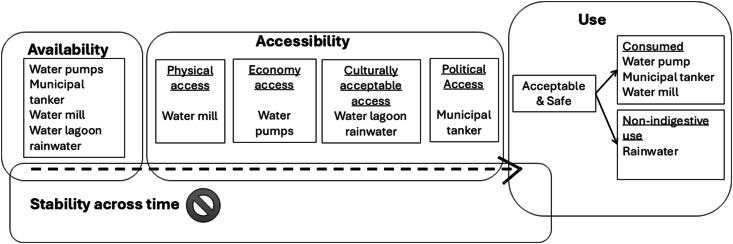
The 4 domains of water security. Adapted from reference [29].

In this context of scarcity, access to available water sources was often constrained by geographic, economic, cultural, and political factors. Consequently, water for consumption was primarily limited to pump water and municipal water, with some participants noting exceptions based on their lack of preference or the presence of a noticeable bleach taste in the water (Figure 4). Although some participants identified rainwater as a potential source of consumption, geographic and environmental conditions, such as proximity to pit latrines and contamination, rendered it unsafe. Among all available options, drilling wells emerged as the most viable method for securing drinking water. The reliability of well water, however, was compromised by multiple challenges, including high temperatures causing pumps to run dry, power outages disabling the pumps, mechanical failures in pump motors, and diminished water flow due to resource sharing with neighbors or hosting visitors. These factors frequently resulted in heightened household water demand, exacerbating the insufficiency of supplies. Consequently, the community’s water stability over time remained uncertain. Thus, taking into account the water security domains in the Pilagá community, we found that *availability* was constituted by the water boreholes, the water provided by the municipality's tanker truck, the community mill, and the lagoon and rainwater; *accessibility* was categorized into physical access (mill in the community), economic access (water drilling), culturally acceptable (water from the lagoon and rainwater), and political access (water from the tanker). As for water use, we were able to observe an *acceptable use* for consumption of water based on community perceptions of taste, clarity, and reliability of supply from the borehole, the municipality and the mill, and the use of rainwater for needs other than consumption. Finally, the critical problem that was evident was the *lack of stability* in water security over time (Figure 4).

## Discussion

Moderate to high levels of water insecurity are a reality for this community and are persistent challenges in the community, with direct impacts on daily life, health and cultural practices. Participants described how limited access to safe water not only affected their physical well-being but also disrupted culturally significant uses of water. These experiences illustrate how water security in this context is shaped by overlapping sociocultural, ecologic and structural factors. Understanding water security among Formosa's indigenous communities is, therefore, critical for both public health and the local economy. Vulnerable populations, such as indigenous groups, face significant water-related challenges [[27], [28], [29]]. Examining water security from a human rights perspective enabled us to explore the multifaceted nature of water insecurity in the Pilagá community, uncovering how structural barriers, resource limitations, and the enduring legacy of colonialism drastically transform lifestyles and severely restrict access to clean water.

Water security, however, extends beyond simply having water availability in a geographic area, as inequitable resource distribution prevents the most vulnerable communities from having access to water. Indeed, for many indigenous families and communities, water security is shaped not only by the availability of water within their territories but, more critically, by the historical and ongoing injustices in resource access and service distribution that affect both the quantity and quality of the water that they have access to [17].

From the perspective of geography, water plays a crucial role in the identity of the Pilagá community, particularly in relation to the Pilcomayo River. The name of the Pilagá neighborhood, “La Bomba” (The Pump), where this study was conducted, reflects the method of water extraction. In the past, families relied on a communal pump to collect water before individual wells were established. Today, households without wells continue to depend on the community pump. Arenas [29] describes water as the key resource that historically dictated the nomadic movement of these groups. According to ethnographic literature on the Peoples of the Gran Chaco, the Pilagá were traditionally nomadic or seminomadic hunter-gatherers, a lifestyle shaped by the spatial distribution of plants. Thus, access to both water and food historically determined their patterns of settlement.

In terms of physical water access, our study identified 2 primary methods: water pumped from wells and delivery by an unreliable and insufficient municipal tanker system. As a result, the community resorted to alternative, unsafe water sources such as rainwater and water from a nearby lagoon—raising significant health concerns. The Pilagá community prioritized physical access to water over concerns for water safety. Although rainwater is generally safer than other alternatives, it is not immune to contamination. The quality of harvested rainwater largely depends on the cleanliness of the collection and storage processes, as well as the effectiveness of management practices. Many individuals are often unaware about nonvisual contamination or cannot afford proper maintenance due to financial constraints or technical incapability [30,31]. In the Pilagá community, this issue is further exacerbated by the observed neglect of storage tank cleanliness, contributing to heightened risks of contamination and health hazards. Studies have shown that air pollution not only renders rainwater acidic and turbid but also introduces heavy metals such as lead into the water supply [32]. Furthermore, rainwater can become contaminated with ash and other pollutants during or after a bushfire [33] a frequent occurrence in the study region during the summer months.

This finding aligns with our observations, where “water access” was understood primarily in terms of availability, rather than quality. This is consistent with research on indigenous communities in Latin America, which similarly lack access to safe drinking water [27,[34], [35], [36]] and experience high rates of diarrhea and gastrointestinal diseases as a result [15,28]. These health issues are frequently associated with the use of pit latrines, as contaminants from excreta can seep into groundwater, significantly increasing the risk of well-water contamination and its subsequent impact on human health [30,37,38]. Studies examining the interaction between pit latrines and groundwater have identified that the presence of fecal contamination in groundwater could lead to outbreaks of waterborne diseases [38,39].

Our findings indicate that the cultural dimensions of water insecurity in La Bomba are shaped by infrastructure, public water policies, which influence perceptions and preferences for what is considered safe water. We showed that the community favored accessing water through pumping from wells and rainwater. In contexts of scarcity, rainwater is often deemed drinkable if properly treated, although most residents are unable to boil it due to the prohibitive cost of fuel [36]. Some of the participants preferred to drink rainwater; we hypothesize that it could be due to this generation valuing more traditional [38] and natural water sources [15]. Socioeconomic and gender inequalities also play a critical role in water access [40], with women bearing the primary responsibility for securing water for their families, a pattern observed in many indigenous communities [15,20]. Globally, women are disproportionately affected by water insecurity, a reality that is unsurprising given the physical demands of water collection [41]. This task not only increases energy expenditure but also limits time available for income-generating activities that could help purchase food [42].

The resulting dynamic adversely impacts the health of women and the well-being of their families. Additionally, the physical and mental strain of securing water contributes to chronic stress. Psychosocial and physical well-being can be adversely affected by concern about diminishing water in the household storage tank or anxiety about contaminants [38]. Among the Pilagá people, feelings of concern, anger, and shame over the lack of safe water reflect these challenges [27,36]. The lack of water significantly disrupts essential daily activities such as bathing, handwashing, cooking, and cleaning, while also exacerbating personal and social challenges. Another study conducted in Kenya found that because of water insecurity, family members faced several challenges, including irregular mealtimes and food shortages, difficulty discussing household or school matters with the mother, frustration over limited bathing and hygiene, and overall dissatisfaction with the absence of an able-bodied family member to help with daily tasks [41].

Water is essential not only for survival but also for health and overall quality of life, with availability, access, and stability as fundamental pillars. Our study found that the most critical domain with water was the lack of long-term stability. Even households with drilled wells lacked reliable access to water due to various influencing factors such as high temperatures in summer causing the motor to overheat and not work, power outages, the well running dry or not enough when household demand increased due to sharing water with neighbors or when households had more visitors. These challenges need to be addressed based on a human rights approach and not just by proposing technical solutions.

These findings highlight the critical importance of addressing water insecurity in sociocultural contexts shaped by both geographic factors, such as the proximity to wetlands like Bañado la Estrella, and historical processes, such as territorial displacement of Indigenous people forcing their nomadic communities to settle permanently in a location. These historical events have had lasting impacts on settlements patterns, access to natural resources, and local water management practices. The application of the HWISE scale not only quantified water insecurity but also illuminated the specific aspects of daily life most profoundly impacted by it. Academically, these results emphasize the necessity of integrating sociocultural factors into analyses of water insecurity and recognizing their reciprocal influence. In practice, these findings have implications for public policies and advocacy for prioritizing the voices and lived experiences of Pilagá community members, mainly of the women while addressing water security challenges in the region. By centering on the perspective of Pilagá culture, such policies can foster sustainable, culturally adequate solutions that endure over time.

Future studies should explore in more depth how historical and environmental factors, such as proximity to wetlands, shape water insecurity in Indigenous communities. Additionally, research should deepen the integration of sociocultural perspectives into water insecurity assessments, particularly by amplifying the voices of women in the Pilagá community. Investigating the long-term effectiveness of culturally informed public policies codesigned with indigenous peoples could also provide insights into sustainable solutions for water security challenges in vulnerable indigenous communities.

### Limitations and future research

We acknowledge several limitations in our study. First, although Pilagá is the native language, the interviews were conducted in Spanish. Although all participants were fluent in Spanish, there is a possibility that certain concepts may not have been fully comprehended when presented in a non-native language. Importantly, although one of the authors belongs to the Pilagá community, the nonindigenous identity of the other authors also shaped the interactions with the community, influencing the dynamics of engagement. In terms of positionality, we approached the study of water security through a Western lens, using a biomedical and biosocial framework to analyze health. This perspective undoubtedly affected our interpretation of the findings. Finally, although the sample size was relatively small, it included representation from every family in the community.

The mixed-methods research design was complex and time-intensive due to the integration of diverse data collection and analysis techniques. Despite the limited sample size, this approach provided depth and context to the data gathered using the HWISE scale, thereby enhancing the study's validity and reliability.

## Author contributions

The authors’ responsibilities were as follows – SIO: designed the study and collected, analyzed, and interpreted the data, drafted the first version of this manuscript and provided final approval for the submitted version; CRV: critically revised the manuscript for important intellectual content and provided final approval for the submitted version; CP: contributed to data collection, analysis, and interpretation and provided final approval for the submitted version; RP-E: critically revised the manuscript for important intellectual content and provided final approval for the submitted version; and all authors: agreed to be accountable for all aspects of the work in ensuring that questions related to the accuracy or integrity of any part of the work are appropriately investigated and resolved.

## Funding

The authors reported no funding received for this study.

## Conflict of interest

RP-E is a Deputy Editor of *Current Developments in Nutrition* and was not involved in the handling of this manuscript. All other authors report no conflicts of interest.
